# Trust, Science Education and Vaccines

**DOI:** 10.1007/s11191-022-00339-x

**Published:** 2022-04-26

**Authors:** Michael J. Reiss

**Affiliations:** grid.83440.3b 0000000121901201 IOE, UCL’s Faculty of Education and Society, University College London, London, UK

## Abstract

The issue of trust in science has come to the fore in recent years. I focus on vaccines, first looking at what is known about trust in vaccines and then concentrating on whether what science education teaches about vaccines can be trusted. I present an argument to connect the phenomenon of vaccine hesitancy to the issue of trust and then argue for what an education about vaccines in school science might look like that takes seriously the notion of respect for students, including students who hold views about vaccination with which science teachers might disagree. Trust in others (people and institutions) varies greatly, both between countries and within countries, and depends on the characteristics of both trustor and trustee, and there are great differences in the extent to which people trust vaccines. However, it is a mistake to think that people who do not trust vaccines are simply ill-informed. There are a range of reasons for rejecting what is often an unexamined narrative about vaccines, namely that vaccines are always desirable. Many people come from communities that have sound reasons for being suspicious of what they are told by governments, business and the medical establishment. COVID-19 and earlier reactions to vaccination health scares show how important high-quality education about vaccines is. Much of that education can take place out of school, but the foundations are laid in school. Vaccine rejection and hesitancy have major global public health implications. Good quality vaccine education should help students understand about relevant biology and the nature of science; it should also be respectful of all students, including those who come from families that reject vaccines or are hesitant about them.

## Context

In this article, I examine how the issue of trust in science is relevant to teaching about vaccines in schools. I began writing it on the day that I was delighted to have received my second AstraZeneca COVID-19 vaccination. Yet, many people are now worried about the safety of this vaccine, and regulators in a number of countries have restricted its use to those over (or under!) a certain age. Initially hailed as a game changer, because of its low cost and easy storage needs, it then faced a series of problems with questions over side effects (Wise, 2021a) and effectiveness (Mallapaty & Callaway, 2021) and disputes within Europe about distribution. A survey undertaken by a reputable polling organisation showed that in March 2021, more people in France, Italy and Spain thought that the AstraZeneca vaccine was unsafe than safe—in France by the remarkable margin of 61 to 23% (Smith, 2021). As Martin McKee, Professor of European Public Health at the London School of Hygiene & Tropical Medicine, said, speaking of the mixed messaging around the vaccine and the way the company communicated information about its trials: ‘This is a company that has taken an innovative product to market in record time but has mishandled communications at every step. Trust and confidence are so important for vaccines – you can’t divorce the two’ (Wise, 2021b: 1). Concerns have not been restricted to the AstraZeneca vaccine. In an international survey undertaken between March and May 2021, there were worries about other makes of vaccine too, with the most commonly cited reasons for not yet having had a COVID-19 vaccine, by those who were eligible for one, being concerns about side effects and whether vaccines had been through enough testing (Imperial College London, 2021). However, the findings of these two surveys differ substantially in a number of regards, indicating how attitudes often change rapidly over time.

In this article, my particular focus is not so much scientific questions about the safety and efficacy of vaccines (cf. Larson, 2020; Oreskes, 2019) but whether we can trust what science education teaches about vaccines. My claim is that there is a link between the safety and efficacy of vaccines, the history of objections to vaccines, public trust in vaccines, current objections by some to vaccines, philosophical arguments (in the context of vaccine uptake) about autonomy and rights, the aims of vaccine education, and the trust that school students have or do not have in what they are taught. This claim is examined and defended in what follows.

Vaccines are sometimes taught in school science as a topic in their own right, often with the ultimate aim of improving public health through enhanced vaccine uptake (Frayon, 2020; García-Toledano et al., 2022). Often, though, they are taught as examples of the application of science, after such topics as the immune system, disease and DNA have been taught. My overall argument is that school science education could do a better job of teaching about vaccines, as has been argued by Dillon and Avraamidou (2020). These authors pose a number of questions for science education in the light of the COVID-19 pandemic, including ‘Is science education research producing knowledge that protects society from catastrophic events’? (Dillon & Avraamidou, 2020: 1), and invite the science education community to respond to them.

At present, although there may well be a range of teacher positions, vaccines are typically presented in school science curricula and textbooks as being unproblematically a ‘good thing’, though, of course, it is individual teachers who interpret curricula and textbooks in their classroom teaching (Ogborn, 2002). These interpretations are important. Both Berkman and Plutzer (2010) and Long (2012) discuss how the beliefs and views of individual teachers about the topic of evolution—another contentious topic—affect how they teach evolution, as it is represented in curricula and textbooks, when it comes to their classroom teaching.

More generally, science teaching needs to consider issues of student diversity (Lee & Luykx, 2006). Yet much school science education fails to consider students who have concerns, or come from families that have concerns, about vaccines, and it fails to prepare all students for situations such as those that may arise long after they leave school when they read or hear conflicting accounts about the safety or general value of vaccines, as in the AstraZeneca case mentioned above.

This is not an article that presents new empirical data, nor is it a formal review of the literature. Rather, it is a conceptual piece. It presents an evidence-based argument that addresses the issue of what students are taught, so as to connect the phenomenon of vaccine hesitancy to the issue of trust. It then argues for what an education about vaccines in school science might look like that takes seriously the notion of respect for students. I maintain that such respect from teachers is needed even if they teach students who hold negative or hesitant views about vaccination with which they (the teachers) disagree. I pay particular attention to COVID-19, given both the significance of the current pandemic and the fact that for many students it is very topical. However, many of the issues about vaccine hesitancy that arise for COVID-19 have previously played a role in distrust of other vaccines, as discussed below. Accordingly, in places I make use of arguments from the history of how the issue of vaccination has been viewed by the public.

My aim is not to castigate school science education but to look at what the science education community (academics and those who determine school curricula, write textbooks, prepare students to be teachers and contribute to formal science examinations) can do to improve and enrich the quality of vaccine education in schools. As I discuss, there are issues to do with how this might be achieved that have commonalities with evolution education and climate change education, where an appreciation of the significance of the nature of science can be of value.

## Trust

This section examines the meaning of trust and goes on to consider the extent to which the general public trusts science and scientists. There has been a long tradition both within science education (e.g. Halliday & Martin, 1993; Sutton, 1992) and more generally (Heidegger, 1962) of examining the origins and use of terms to help explore how they are understood and used. In English, the word ‘trust’ exists both as a verb and a noun. The word comes from the Old Norse *traustr*, meaning ‘strong’, ‘safe’ or ‘reliable’ (Oxford English Dictionary, 1971). Various dictionaries agreed that the trust nowadays entails the acceptance of the truth of a statement without further evidence or investigation, so that if I consider you trustworthy, I am more likely to accept what you state to be the case than if the same statement is made by someone whom I consider to be less trustworthy. While the meanings of words depend more on how they are used than on formal definitions (Wittgenstein, 1953), trust has been defined as ‘the probability that [someone] will perform an action that is beneficial or at least not detrimental to us is high enough for us to consider engaging in some form of cooperation with [them]’ (Gambetta, 1988, p. 217). If I trust you, I am less likely to make checks to ascertain the veracity of what you tell me; I am more likely to assume that you will not act against my interests. While reality is important in the determination of trust, so too are judgements and perceptions; there is therefore a social dimension to trust.

The academic literature on trust divides into the conceptual and the empirical. One long-standing strand of conceptual writing is theological, deriving from considerations of πίστισ (*pistis*) in the Christian scriptures (the New Testament), a word also translated as ‘faith’ through its usage in secular Greek of the time to mean ‘reliability’, ‘fidelity’, ‘assurance’ or ‘pledge’. Interestingly, in the Septuagint, the third and second BCE translation into Greek of the Hebrew Bible (the ‘Old Testament’ to Christians), *pistis* probably never means ‘faith’ or ‘trust’ but something closer to ‘firmness’ or ‘assurance’ (Howard, 1974). It may therefore be that the sense is to do with changing one’s mind, something directly relevant to people’s views about vaccines.

In the broader and generally more recent social science literature, it has been argued that trust (i.e. as placed by a trustor in a trustee) can be distinguished from confidence, in that confidence is more about a belief in the competence of the trustee. A breakdown in trust may be more easily repaired if the trustor interprets the reason for the breakdown as a failure in the trustee’s competence rather than their honesty or benevolence (Nooteboom, 2017). Tschannen-Moran (2017) writes about the ‘growing awareness of the crucial role that trust plays in every aspect of a school’s functioning and especially to student outcomes’ and argues that it rests upon ‘confidence in the other party’s benevolence, honesty, openness, reliability, and competence’.

Fortunately, the word ‘trust’ is widely understood, both in English and in other languages—though this is not to minimise the issues involved in measuring it empirically, for example through surveys (Miller & Mitamura, 2003). Trust in others (people and institutions, including the various institutions—e.g. universities, government research institutes and companies—that fund and/or undertake scientific research and development) varies greatly, both between countries and within countries, and depends on the characteristics of both trustor and trustee. For instance, in Norway, Sweden and Finland, more than 60% of respondents in the World Value Survey think that people can be trusted, whereas in Colombia, Brazil, Ecuador and Peru, less than 10% think that this is the case (Ortiz-Ospina & Roser, 2016). These differences seem, at least in part, to be the product of nation-specific histories. In the UK, Table 1 shows trust in different types of people. Nurses, doctors, engineers, teachers and scientists all do very well, which might encourage us as science educators. Worldwide, 73% of people would trust a doctor or nurse more than any other source of health advice, including family, friends, religious leaders or famous people, and 72% of people trust scientists—even though 57% do not think they themselves know much, if anything, about science (Wellcome Trust, 2019). Worldwide, levels of trust in science, in scientists and in doctors and nurses all increased during the first year of the COVID-19 pandemic (Wellcome Trust, 2021). A pre-COVID study (Hamilton et al., 2015) showed in the USA that Democrats were substantially more likely than Tea Party (particularly right-wing Republicans) supporters to say that they trusted scientists for information. Subsequently, during COVID, vaccination rates in the USA have been substantially higher among Democrats than Republicans (Albrecht, 2022).

**Table 1:** Trust varies greatly depending on the characteristics of the trustor (e.g. their country of residence) and the trustee (e.g. their profession). These data show the responses to Ipsos MORI of 1873 British adults aged 18 + in October 2020 who answered ‘yes’ when asked ‘Now I will read you a list of different types of people. For each would you tell me if you generally trust them to tell the truth, or not?’ (Clemence, 2020)

Type of person	Trust
Nurses	93%
Doctors	91%
Engineers	89%
Teachers	85%
Judges	84%
Professors	83%
Scientists	82%
Museum curators	82%
Care home workers	76%
Home delivery drivers	75%
The police	71%
Lawyers	61%
Civil servants	60%
The ordinary man/woman in the street	57%
Clergy/priests	56%
Economists	53%
Pollsters	53%
Television news readers	50%
Trade union officials	49%
Bankers	44%
Local councillors	42%
Landlords of private residential properties	37%
Business leaders	33%
Professional footballers	30%
Estate agents	27%
Journalists	23%
Government ministers	16%
Politicians generally	15%
Advertising executives	13%

A distinction can be made between the public’s trust in the methods of science and in those who are responsible for science being undertaken (scientists, governments, industries and other funders). While support for the principles and methods of science is generally high, that in scientists and funders can be lower (Huber et al., 2019). Scientists (by which I mean natural scientists, whether working for governments, in universities or in the private sector) are not perfect. They not infrequently stick to favoured theories longer than the evidence strictly warrants (Kuhn, 1970), and they sometimes manipulate their findings inappropriately (Briggs & Reiss, 2021). There is an increasingly realisation that scientific plagiarism and even fraud are more frequent than has generally been acknowledged (Reydon, 2020), which contributes to undermining trust in science. It is very difficult to determine how common fraud in science is. One study found that 2% of scientists admitted to have fabricated, falsified or modified data or results at least once and argued that this figure was probably an underestimate (Fanelli, 2009).

The issue of trust in science and scientists is relevant to the question of trust in vaccines and vaccine education as vaccines are an example of applied science. The next section looks at the issue of trust in vaccines and at why some people reject vaccination.

## Trust in Vaccines

Much less is known about the perceptions and decision-making processes surrounding vaccination in school students than in their parents and the general public (Sandler et al., 2020), though research on school students is beginning to increase in the light of COVID. In a US study of 9th graders, Willis et al. (2021) found that only 42% reported they were not hesitant at all about getting a COVID-19 vaccine, 22% were a little hesitant, 21% were somewhat hesitant and 15% were very hesitant. A comparable study of 9–18-year-old school students in England found that 50% said they would opt-in to take a vaccination, 37% were undecided and 13% said they would opt out (Fazel et al., 2021). It is clear that vaccine hesitancy among school-aged students can be high. Fazel et al. (2021: 1) explicitly note that ‘There were indications that those students who would opt-out had higher levels of marginalisation and mistrust’.

Vaccines are one of medicine’s great success stories, preventing (pre-COVID) some two to three million deaths a year (World Health Organization, 2019). Yet vaccination rates were falling even before COVID-19 arrived on the scene, in part because of the now refuted suggestion that autism rates were rising as a result of vaccination against measles, mumps and rubella (MMR) (Flaherty, 2011). In 2019 the World Health Organization identified vaccine hesitancy as one of the ten threats to global health, with measles, for example seeing a 30% increase in cases (World Health Organization, 2019). One major study (again, undertaken pre-COVID) found that between November 2015 and December 2019, public confidence in the importance, safety or effectiveness of vaccines fell in Afghanistan, Azerbaijan, Indonesia, Nigeria, Pakistan, The Philippines, Serbia and South Korea (de Figueiredo et al., 2020). There are a variety of reasons for this. For example, in the Philippines and Indonesia, vaccine confidence plummeted in 2017 when the vaccine manufacturer Sanofi announced that its newly introduced dengue vaccine posed a risk to individuals who had not previously been exposed to the virus; in South Korea, an online community named ANAKI (Korean abbreviation of ‘raising children without medication’) strongly argued against childhood immunisation at this time, which dented public confidence.

Vaccination began when Edward Jenner famously vaccinated 8-year-old James Phipps in 1796 with extracts from cowpox lesions obtained from the dairymaid Sarah Nelms, though there had previously been a long history of variolation (in which material was taken from someone who had smallpox and then introduced into the person being treated, by scratching the skin or via a nostril). The success of vaccination in preventing smallpox meant that the practice of vaccination soon gained in popularity and spread. However, objections to vaccination against smallpox did not take long to arise. There were concerns about whether vaccination could be trusted, specifically about its safety and efficacy. Of course, it is in the nature of life that no technology can ever be guaranteed to be totally safe or effective, but it is hardly a surprise that early manufacturing practices for vaccines did not come up to the standards that are required nowadays. In the case of smallpox, even in the 1960s, about one in three people given the vaccine had to take days off work or school because of their reactions to it, and about one in a million people died (Belongia & Naleway, 2003).

It is not surprising that many people, when vaccination began, were suspicious about the practice of infecting people with a disease (cowpox), even if the argument was that this would prevent a far more serious disease (smallpox). A very different objection to vaccination, one to do not with its efficacy but focusing on human freedoms, arose once it began to be mandatory. The purpose of the UK 1853 Compulsory Vaccination Act was as indicated by its title. The Act required children to be vaccinated within 3 months of their birth (4 months for orphans); parents or guardians who failed to comply, without sufficient reason, were liable to a fine of £1 (a very large sum for most people at the time). This policy helped reduce the incidence of smallpox considerably but was controversial and deeply resented by some. As the anti-vaccinationist, John Gibbs, put it in 1854: ‘Are we to be leeched, bled, blistered, burned, douched, frozen, pilled, potioned, lotioned, salivated by Act of Parliament’? (Durbach, 2000: 45).

No doubt there were those who objected to vaccination being compulsory and who simultaneously held that vaccines were unsafe or ineffective, but in principle, one could (and can) object to mandatory vaccination even if one believes that they are effective and safe. In such a case, it is not that one is distrustful of a vaccine’s efficacy and safety but that one rejects and does not trust attempts to make its use mandatory. This is an argument about autonomy and rights; a utilitarian perspective might argue that it is acceptable for vaccination to be made mandatory so long as the overall benefits are sufficient to outweigh the concerns of the minority opposed to such compulsion. Nowadays, countries vary considerably in the extent to which vaccination is mandatory, and when it is, this is typically only for certain categories of individuals, e.g. travellers, health professionals and those attending nurseries, preschools or kindergartens (Vaz et al., 2020).

**Fig. 1:**
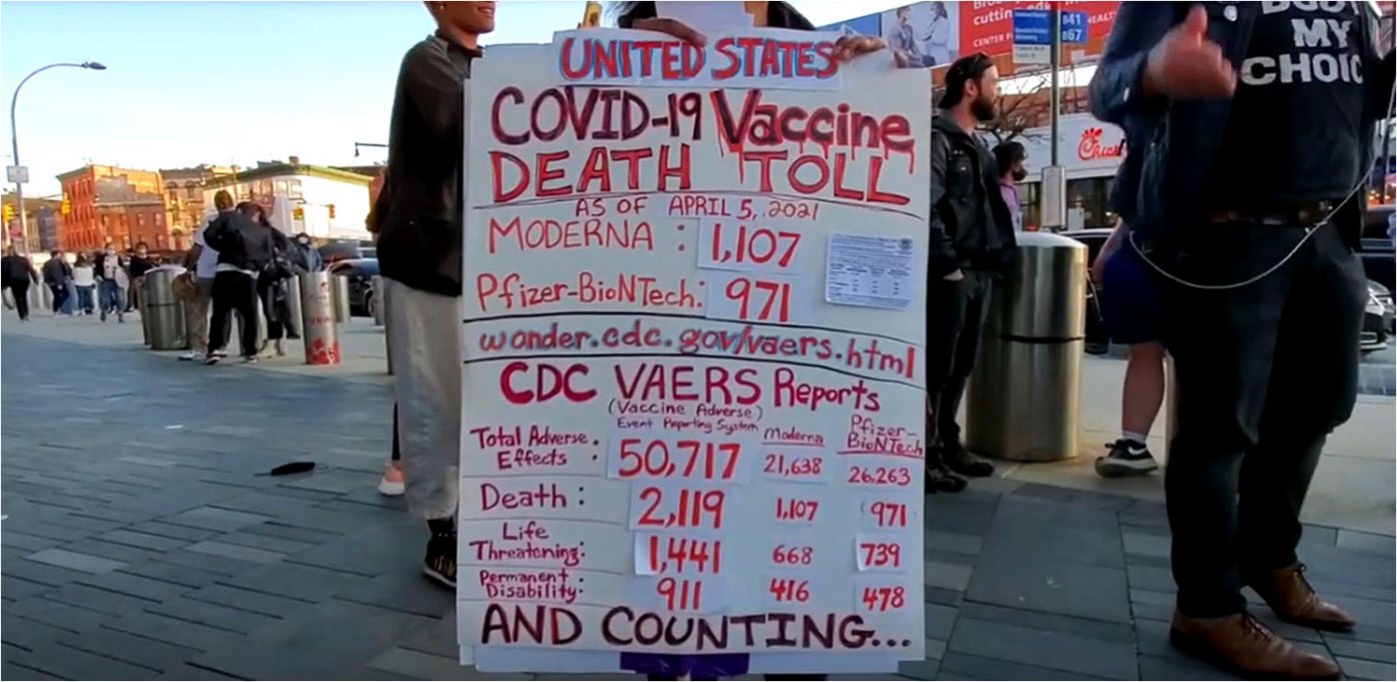
Anti-vaccination protestors at the Barclays Center in Brooklyn, New York, on 5 April 2021 where no one was allowed in to see the basketball game without proof of vaccination against COVID-19. Taken by Felton Davis https://commons.wikimedia.org/wiki/File:21-04-05_03_Vaccine_Protest_at_Barclays_(51106744481).jpg

As more and more diseases were controlled by vaccination development (including whooping cough in 1914, diphtheria in 1926, tetanus in 1938), objections to the practice diminished. In the USA, by the mid-twentieth century, one authority concluded, ‘With the improvements in medical practice and the popular acceptance of the state and federal governments’ role in public health, the anti-vaccinationists slowly faded from view, and the movement collapsed’ (Kaufman, 1967: 478). However, this did not last, as illustrated by Fig. 1. Today’s objections to vaccinations have similarities with those of the past—vaccines do not work, they are not safe, they infringe personal liberties—but have colonised new areas. Additional moral objections have arisen, and, in part because of the rise of conspiracy theories, there is in a number of countries greater scepticism now about government advice and the activities of big business, including pharma (Foster & Frieden, 2017; Miller, 2013).

One moral objection to certain vaccines has arisen because of the historical use of aborted foetuses in their manufacture. Several live vaccines against rubella (Meruvax, Rudivax, MR-VAX) and vaccines against hepatitis (A-VAQTA and HAVRIX), chickenpox (Varivax) and poliomyelitis (Polivax) fall into this category (Pelčić et al., 2016), and the Roman Catholic Church has suggested that these vaccines should be avoided (Pontifical Academy for Life, 2006). This argument has resurfaced as the Johnson & Johnson vaccine against COVID-19 similarly uses cell lines from aborted foetuses (*not* foetal cells). The Roman Catholic Archdiocese of New Orleans advised that if the Moderna or Pfizer vaccine is available, Catholics should choose to receive either of those rather than the Johnson & Johnson vaccine because of its extensive use of abortion-derived cell lines (Archdiocese of New Orleans, 2021).

Again, as with objections to compulsory vaccination, the issue here is not to do with vaccine efficacy or safety. Rather, the objections stem from the fact that many Roman Catholics, in common with many other Christians, some people of other religions and some people of no religious faith, hold that the very large majority of elective abortions are intrinsically wrong. There can be certain exceptions; for example, the principle of double effect means that if an abortion is undertaken with the primary intention of saving the mother’s life, then the consequent loss of a foetus’ life can be morally acceptable within the Roman Catholic tradition. In fact, despite the Roman Catholic teaching that the very large majority of elective abortions are morally unacceptable, there are a range of views held within Roman Catholicism, including at very senior levels, about the acceptability of vaccines that rely on the use of such abortions (Millies, 2021). There are, of course, ethical arguments in favour of abortion, of which perhaps the principal one is the woman’s right to choose, along with the fact that prohibition of abortion can result in more women dying as a result of clandestine abortions (e.g. da Silva, 2009). The general point is that for many religious people if push comes to shove and they feel that science and their religious faith are in tension, they are more likely to trust the teachings of their religion and to act on them (O’Brien & Noy, 2018).

Non-religious objections to vaccines stem from a number of sources. In particular, some communities, especially black and minority ethnic communities, have historically had good reasons not to trust what governments, big business or even the medical establishment say to them. For example, the notorious Tuskegee Syphilis Study ran for 40 years. It only ended in 1972, thanks to a whistleblower, Peter Buxtun, who leaked information about it to the *New York Times* which then published the story on their front page (McVean, 2019). It transpired that treatment for black patients with syphilis had systematically been withheld for decades, long after antibiotic treatment was available and despite the individuals concerned being told that they were being treated, all this in order to study the ‘natural history’ of untreated syphilis. No one was ever prosecuted.

More recently and specifically in relation to trust around vaccinations, it transpired that in its attempts to locate Osama bin Laden in the wake of the September 11 attacks in the USA in 2001, the Central Intelligence Agency (CIA) used a fake hepatitis B vaccination project to collect DNA in the neighbourhood in Abbottabad, Pakistan, where he was hiding (Anon, 2013a, b; Martinez-Bravo & Stegmann, 2021). The intention was to obtain DNA from bin Laden’s children to confirm bin Laden’s presence (bin Laden’s sister had died in the USA in 2010, and her DNA was available to the US authorities), thus allowing an expensive and dangerous mission to proceed, with the intention of assassinating bin Laden, as indeed was done.

News of the CIA initiative led to attacks on polio vaccination workers in Pakistan, with legitimate health care workers targeted as US spies, and some 70 killed. As a consequence of the fatalities, organisations such as the UN suspended polio vaccination efforts in Pakistan, and parents refused to have their children vaccinated. The Pakistani Taliban launched an anti-vaccine propaganda campaign, maintaining that the polio vaccination campaigns were a conspiracy to sterilise the Muslim population. The result was an upsurge in polio cases in Pakistan.

### Trust in COVID Vaccinations

We have only had COVID vaccinations for a short period of time. Inevitably, therefore, we do not have data on their long-term effectiveness and safety. Assurances about these are necessarily extrapolations from data gathered on other vaccines, informed by the absence of a known mechanism whereby COVID vaccinations might prove dangerous. In a qualitative study undertaken to investigate vaccine hesitancy during the COVID-19 pandemic, in-depth interviews were undertaken in Bradford, a part of the UK that is characterised by high deprivation and ethnic diversity and above-average rates of COVID-19 (Lockyer et al., 2021). The huge amounts of information surrounding COVID-19 had left many of the interviewees feeling overwhelmed and confused. As one person said:

The government aren’t being clear and they’re saying one thing but then they’re saying other things, and basically what they’re trying to do, they’re trying to please everybody all of the time, it doesn’t happen. (Male, 45–54, Asian or Asian British) (Lockyer et al., 2021: 5)

A number of interviewees were concerned at how rapidly the COVID-19 vaccines had been produced, believing that side effects could not yet be known. One interviewee (Male, 35–44, Asian or Asian British) said he wanted to wait 3 to 6 months to see what the effects were on others. Another interviewee said:

People are saying they don’t know how safe it is, plus they’ve made it so quick we don’t know the side-effects it’s going to have in the future. I mean it’s probably safe because they wouldn’t be allowed obviously to give it to us otherwise, or maybe they would you know, sometimes they don’t care, but you just don’t know if it could cause infertility, it could cause cancer in the future. (Female, 25–34, White British) (Lockyer et al., 2021: 7)

A number of interviewees reported rumours that certain communities were being targeted to test vaccination against COVID-19:

I think what the community are saying is that the vaccine is testing people, they’re just using people as the guinea pigs ... we experience discrimination for many years, and if we've been focused for, if the Slovakian authorities we are focused especially on the Roma, and the focus is they will be testing them because they were noting who could be spreading all this coronavirus, they may think the same thing now why are we going to offer immunisation, because they’re going to trial it out on us. (Male, 35–44, White Other – Gypsy or Irish Traveller) (Lockyer et al., 2021: 7)

The importance of trust is indicated by the conclusions reached by the authors of this study:

Vaccine hesitancy could be attributed to: safety concerns, negative stories and personal knowledge, all of which had been amplified by recent exposure to misinformation via social media. We found that the more confused, distressed and mistrusting the participants felt during COVID-19, the more likely they were to be hesitant about uptake of the COVID-19 vaccine. (Lockyer et al., 2021: 8)

These reasons for vaccine hesitancy are echoed in other studies conducted elsewhere (e.g. Hacquin et al., 2020; Wang et al., 2021). Jones et al. (2021), in a qualitative study with 16–29-year-olds (the most vaccine hesitant age group) in the UK, found that vaccine hesitancy was due to distrust of vaccines on the grounds of safety concerns, distrust of government and of those encouraging vaccine take up, concern about known and unknown side effects (including on fertility) and belief it was unnecessary for those at low risk of harm from the virus. It is also known that those who are more likely to believe in conspiracy theories—such as that the COVID-19 vaccine is a cover for implanting trackable microchips—have lower levels of trust in institutions (Ipsos, 2021). In the next section, I build on the issues of trust in vaccines and vaccine hesitancy to consider what might be the aim of vaccine education, particularly in school science teaching, and argue that there is a place for better education about the nature of science.

## Vaccine Education

This section looks at what good quality vaccine education in schools might entail and what we might hope from it. The argument is based on the assumption that school science education should benefit all learners (Alberts, 2009) and should be respectful of students even if they do not hold mainstream scientific views (McKinley et al., 1992; Reiss, 2019). For a long time, it has been widely assumed by many people in authority (though not all—e.g. Jair Bolsonaro, Donald Trump) that vaccines are unquestionably ‘a good thing’, e.g. the World Economic Forum (Deshpandé et al., 2021). Too often it has been presumed that those who hesitate about vaccines or even reject them are either ill-informed or selfish (relying on others to vaccinate themselves or their children so that herd immunity—a goal of most vaccination programmes—is achieved) (Rozbroj et al., 2019). This approach fails for two reasons: first, on instrumental grounds—treating learners as ill-informed or selfish is not particularly conducive to their learning—and, secondly, because it is intrinsically disrespectful to people, something that in most countries is politically unwise and conflicts with certain major moral frameworks, such as Kantianism.

Vaccine hesitancy around COVID-19 and earlier reactions to vaccination health scares show how important high-quality education about vaccines is. Much of that education takes place out of school, but the foundations are laid in school, and school education is important for vaccine education for many reasons—including the amount of time that people spend in schools and the fact that much school science education is undertaken by trained professionals.

A school curriculum for vaccine education needs to take history seriously. At present, school vaccine education too often gives the impression that everything gets better over time: originally there were lots of horrible diseases; thanks to the advances of science, we first found a vaccine for smallpox and then for many other infectious diseases; if only everyone would get their children vaccinated, millions of lives would be saved. Equally, curricula that include the topic of vaccination need decolonising as there is a danger of giving the impression that everyone sat around producing large numbers of babies, expecting many of them to die, until Western medicine arrived (particularly, vaccinations—Jenner, Salk and others—and antibiotics), thus enabling people to be healthier and start having smaller families.

Of course, a good school vaccine education should take science seriously and teach it well. However, there is more to the science of vaccination than is sometimes supposed. Obviously, there is basic physiology—the way in which the body’s immune system reacts to pathogens and the way in which vaccination mimics and stimulates this to reduce the likelihood of subsequent infection. But there is also an evolutionary perspective in which there is something of an arms race between the immune system and pathogens, with uncertain outcomes (Anderson & May, 1991) with regard to changes in infectiousness and severity over time. Then there are the increasingly diverse ways in which vaccines can be produced, each of which has its own advantages and disadvantages. Here, as so often is the case within school science, we are talking about the applications of science, namely technology. Technology is treated in some countries as a separate school subject but, in other countries, lumped with science, even though it has been argued that the two subjects differ greatly with respect to their ontologies and epistemologies (Barlex et al., 2020; Matthews, 2014) and have a complicated relationship (Ziman, 2000).

As far as vaccine education goes, there is much valuable science learning that can result from students learning how vaccines are produced. In the case of vaccines against COVID-19, multiple approaches have been used: peptides, virus-like particles, viral vectors (replicating and nonreplicating), nucleic acids (DNA or RNA), live attenuated virus, recombinant designed proteins and inactivated virus (Shahcheraghi et al., 2021). Each of these approaches has advantages and disadvantages. Students could usefully learn why such a range of approaches are used (new approaches are currently coming on the market, and it is too early to know if they will supplant existing approaches or not; different approaches may be better suited to different countries, depending, for example, on vaccine storage facilities, etc.). In this way, students can be helped to realise that the vaccine science they learn in their classrooms, in addition to being intrinsically worth learning, directly connects to issues of vaccine efficacy.

In terms of the science of vaccines against infectious diseases, it should not be thought that even determining the numbers who die from a particular disease is straightforward (Reiss, 2020). For example, in the case of COVID-19, it is clear that many countries under-report deaths resulting from it (Whittaker et al., 2021). Some of the reasons for this are to do with a lack of capacity and some are overtly political (as has been widely reported in certain countries that are keen to minimise the significance that COVID-19 has had and is having, whether to do with attempts to reduce internal dissent or to continue to attract foreign visitors) but others are to do with more fundamental issues to do with scientific measurement (Reiss, 2020). For a start, attributing cause of death is often a matter of judgement even if we possess perfect knowledge about the circumstances of a person’s death. For instance, just because after my death I am shown by testing to have had COVID-19 does not necessarily mean that I died because of COVID-19 infection. I might have died of pneumonia, though it might have been the case that had I not had COVID-19, I would have been more likely to have recovered from pneumonia. Students could be encouraged to look at data on COVID-19 fatalities and compare the different ways in which such data are determined. For instance, does being categorised as a COVID-19 fatality require a pre-mortem test for COVID-19, and if it does must this be a PCR (polymerase chain reaction) test or is a lateral flow test adequate? If a COVID-19 infection is diagnosed on clinical grounds, to what extent do symptoms serve to distinguish COVID-19 from other so-called respiratory infections? How useful are ‘excess death’ calculations (Woolf et al., 2020)?

The topic of vaccination also provides a wonderful way of helping school students better understand aspects of the nature of science—which can be understood as encompassing what science is, how it is undertaken and the fact that, while reliable, it is a human endeavour and its findings are always open to revision (Erduran & Dagher, 2014; Lederman, 2007; McComas, 2020). Through a range of teaching approaches, including examinations of historical case studies, debates and the use of argumentation, students can be helped to think critically about vaccination *and* to deepen their understanding of the nature of science. One of the shortcomings of school science education around the world is that too few students leave their schools realising that science is both a set of ways of establishing robust knowledge about objective aspects of the world and a body of such knowledge. Furthermore, few school students appreciate that the confidence that can be placed in scientific conclusions varies, depending on the nature of that knowledge (Deng et al., 2011). While science operates in both cases, we can use Newton’s laws when calculating the trajectories of projectiles with far greater confidence than we can use our knowledge of human behaviour and immunity when deciding what recommendations to give about mask wearing or social distancing so as to prevent COVID-19 transmission.

Students can (should) also be helped to appreciate that the role of science (taken as the natural sciences) in addressing objections to vaccination is more limited than is sometimes thought. Objectors who cite concerns about safety and the effectiveness of vaccines are typically not saying that a cost–benefit analysis on the grounds of efficacy or safety comes down against the use of vaccines—indeed, such cost–benefit analyses very strong support vaccine efficacy and safety. Rather, objectors may be saying that vaccines do not *always* work and are not *totally* safe. You cannot argue against these objections on scientific groups—a lesson that should have been learnt from the widespread rejection of genetically modified foods in some countries (Reiss & Straughan, 1996).

Furthermore, consider the objection that vaccines are often made in ways that are morally unacceptable. It all comes down to what one means by ‘morally acceptable’. For example, as discussed above, a number of widely used vaccines use cell lines derived from foetuses that were electively aborted decades ago. While for many people elective abortions (i.e. terminations rather than miscarriages) are, at least in certain circumstances, permissible, for many other people, they are not, often on religious grounds. These differences of opinion—deeply held convictions—cannot be reconciled by any method of science (Reiss, 1999). They simply lie out with science, being situated in the domain of moral philosophy or values more generally; many people simply approach these issued from very different perspectives—they occupy different worldviews (Matthews, 2009).

The discipline of moral philosophy, whether examined within the frameworks of consequentialism, deontology or virtue ethics, intersects with vaccination policy and practice in a number of respects (e.g. Nuffield Council on Bioethics, 2021). For a start, consider the issue of the distribution of vaccines when they are in short supply. Even setting aside international issues to do with vaccine nationalism, should, for example, those who are most susceptible to the disease in question be favoured (e.g. in the case of COVID-19, the elderly, those with underlying health conditions, those who are overweight, men, those in certain occupations and those from ethnic minorities), those who would do the most public good if vaccinated early (and who decides who these are—most would agree with respect to health and care workers but what about members of the police?), or those who would have the most years of good quality life left (leading to a consideration of QALYs—quality-adjusted life years)? Other ethical issues of vaccination include whether vaccinations should be mandated or strongly encouraged (for example by making them a condition of public schooling or access to sporting events or restaurants), the validity of religious and other objections to vaccination (including a discussion as to whether this is comparable to conscientious objection to military conscription or eating certain foods), health care rationing and whether we have a duty to be vaccinated (or to vaccinate our children).

Mention of all the ethics teaching about vaccines that could take places leads on to the question as to where vaccine education should take place in schools. While science lessons are and are likely to remain the main place in which teaching about vaccination takes place in schools, there is an argument that other subjects, including geography, history, philosophy (often taught in my country in religious education lessons) and mathematics, might play a part—the last of these raising more general issues to do with applied mathematics and the choice of examples/contexts in mathematics teaching. For example, exponential growth/decay is presently more likely to be taught through radioactivity than R numbers, even though the latter is likely to be more engaging to some categories of learners, while variations in R numbers mean that certain aspects of exponential growth/decay can be more straightforwardly taught through population growth/reduction than through radioactive decay. School mathematics through the context of disease and vaccination might also help students to develop a better understanding of risk, of charts and graphs, of a range of functions (including linear and exponential ones), of the relationship between a function and its gradient (e.g. the numbers and rates of change of infections, hospitalisations and deaths) and of mathematical modelling in attempts to predict the consequences of changes in input variables (e.g. the frequency with which infected people meet new people) on output variables (e.g. new infections).

As always, good teaching is helped by a teacher surveying the views of their students and using these to inform their teaching. It is also the case that science lessons themselves can make more use both of interdisciplinary teaching and of the various approaches, such as discussion, role play and debate, that are typically more often found in some other school subjects. There is evidence that such teaching can be more motivating for many students and lead to better learning (You, 2017). It may be that such teaching proves efficacious for the approach to vaccine education advocated here, which treats it as a controversial issue, in the everyday sense of the term—as ‘controversial’ is used when discussing the theory of evolution or anthropogenic climate change, i.e. not that there are deep controversies among scientists about the fundamentals (of course, there are always scientific controversial at the frontiers of science) but that it is contentious for a significant proportion of the general public.

Allied to the approach of treating vaccines as controversial is to suggest that vaccine education would benefit from cultural competence in teaching. The concept of cultural competence is rarely discussed in the context of school science education. However, it is prevalent in the context of extra-school health education (e.g. Jeffreys, 2015). For example, Seeleman et al. (2009: 229), in the context of medical education, argue that cultural competence entails:

*knowledge* of epidemiology and the differential effects of treatment in various ethnic groups; *awareness* of how culture shapes individual behaviour and thinking; *awareness* of the social context in which specific ethnic groups live; *awareness* of one’s own prejudices and tendency to stereotype; *ability* to transfer information in a way the patient can understand and to use external help (e.g. interpreters) when needed, and *ability* to adapt to new situations flexibly and creatively.

Cultural competence is therefore a pedagogical approach that is comfortable with learner diversity and respects individuals with a range of views. It would be good to have science education studies that researched the effects of a range of school teacher pedagogies on what students get from their vaccine education, including students who reject or are hesitant about vaccines. What we want, I would argue, are teachers who are respectful of all their students but still teach them about the safety of vaccines and their role in public health.

## Conclusions

The issue of student trust in their school education is under-researched, with the emphasis in the research that has been undertaken mainly being on trust between students and their teachers (Platz, 2021), which is not the focus of this article. However, we do know quite a lot about the way in school science education, especially as students age, fails to engage with many of them (Archer et al., 2012; Sheldrake et al., 2019). When it comes to certain long-standing contentious topics, such as evolution and climate change, we know more than we do about vaccines with respect to how many people reject them, though the data are thinner for school students than for college students and other adults (Long, 2011; Reiss, 2018; Dawson & Carson, 2020).

Vaccinations have already saved the lives of literally hundreds of millions of people, and yet an increasing number of individuals are hesitant about vaccination, either for themselves or their children. Schools have long taught about vaccines, and almost any analysis of the purposes of science education would cause us to want more young people to understand about vaccines by the time they leave school. However, trust is an important issue both for vaccines in general and for vaccine education in particular. A vaccine education that students and their parents trust will be one that does not castigate those who are hesitant about vaccines.

Students have misconceptions about vaccination and the immune system (Carson et al., 2018) as they do about most scientific topics. These misconceptions include such things as the nature and size of microorganisms, how vaccines operate and the workings of the immune system. However, there is more to vaccine education than conceptual change and the correction of misconceptions, important as these are. There is much to be said for vaccination being considered in school science education as a socio-scientific issue that addresses social and ethical issues and is for some students of sufficient personal significance that it would benefit from sensitive teaching (Reiss, 2019). In particular, it should not be assumed that simply teaching more about the facts of vaccines will lead to more people accepting them, as this is not necessarily the case for other controversial science topics (Drummond & Fischhoff, 2017). However, and encouragingly, there is evidence that greater knowledge of the nature of science and a more mature view of how to mitigate scientific disagreements each relate positively to acceptance of evolution, climate change and vaccines (Weisberg et al., 2021).
